# Community Feeling and Narcissism as Two Opposite Phenomena

**DOI:** 10.3389/fpsyg.2020.515895

**Published:** 2020-10-27

**Authors:** Alina Kałużna-Wielobób, Włodzimierz Strus, Jan Cieciuch

**Affiliations:** ^1^Institute of Psychology, Pedagogical University of Kraków, Kraków, Poland; ^2^Institute of Psychology, Cardinal Stefan Wyszynski University in Warsaw, Warsaw, Poland; ^3^URPP Social Networks University of Zurich, Zurich, Switzerland

**Keywords:** community feeling, grandiose narcissism, narcissistic admiration and rivalry, vulnerable narcissism, social interest

## Abstract

The objective of the current study was to examine the relations between narcissism and Adler’s community feeling. Based on theoretical considerations, we claim that community feeling can be treated as an opposite pole of narcissism and we expected that: (1) both grandiose and vulnerable narcissism would be negatively related to community feeling and that (2) grandiose and vulnerable narcissism would be positively related to anti-community domination and isolation. A sample of 520 university students (M_age_ = 21.37, SD_age_ = 4.31) completed the Community Feeling Questionnaire (CFQ), the Narcissistic Admiration and Rivalry Questionnaire (NARQ) and the Hypersensitive Narcissism Scale (HSNS). Structural equation modeling largely confirmed our expectations. These results suggest that narcissism can be understood in terms of a deficit in community feeling. It turned out that community feeling and narcissism are related constructs but they are not reducible to each other.

## Introduction

In the first half of the 20th century, two great analysts, the creator of psychoanalysis – Sigmund Freud and the creator of individual psychology – Alfred Adler, independently described two seemingly related constructs. On the one hand, Freud (1914/1955) described the phenomenon of (secondary) narcissism, which was considered to be negative and maladaptive. On the other hand, Adler (1938/2011) described the phenomenon of community feeling, a positive, pro-health disposition, which is beneficial from the viewpoints of both individuals and society. Interestingly, the characteristics of people demonstrating a lack of community feeling resembles the characteristics of narcissistic people. Additionally, modern narcissism research distinguishes two forms: grandiose and vulnerable narcissism (Wink, 1991; Pincus et al., 2009, Miller et al., 2012; Krizan and Herlache, 2017). Similarly, a lack of community feeling can manifest in two ways: the pursuit of domination and anxious isolation (Kałużna-Wielobób, 2017). The current study examines the relationship between two forms of narcissism and community feeling including the two anti-community orientations.

### Community Feeling

Community feeling (or social interest: germ. *Gemeinschaftsgefühl*) was described by Alfred Adler in the 1930s based on data collected by him in psychotherapeutic practice (case studies, clinical research). Below we present the understanding of community feeling proposed by Adler in his classical texts (Adler, 1935, 1938/2011, 2005). According to Adler (1935, 1938/2011, 2005) community feeling can be treated as a relatively stable individual characteristic (personality disposition) throughout life. It refers to one’s dominant life motivation and the basis for human connectedness which is both a sense of unity and harmony. People with a high sense of community feeling are motivated by the pursuit of the common good. Caring about the common good, they strive to make their actions beneficial both for others and for themselves, and consider the effects in different time perspectives – the present and the near future, but also effects for future generations. They have a strong sense of bonds, in contrast to people low in community feeling who are egocentric and may feel alienated and isolated. People low in community feeling may aim to show their superiority over others, confirming their value by being better than others and acquiring a dominant position, which will compensate for their feelings of inferiority. People low in community feeling primarily focus on personal benefits, without regard for the welfare of others or the common good. They try to overcome their inferiority complex by striving to achieve successes that will show their superiority over other people or withdraw from activities in which they do not expect to be successful, which would raise their sense of self-worth. Community feeling is a disposition with far-reaching consequences that are visible in many domains. Table 1 presents the characteristics of people high and low in community feeling, based on Adler’s (1938/2011, 2005) texts, including the most important spheres and aspects in which community feeling manifests itself.

**TABLE 1:** Characteristics of high and low community feeling based on Adler’s (1938/2011, 2005) texts.

**Sphere/aspect**	**High community feeling**	**Low community feeling**
Dominant life motivation	Acting toward the common good (also considering future generations).	Acting toward overcoming other people, being “better than others,” achieving a dominant position.
Self-esteem	Feeling of self-worth flowing from what one has to offer, adequate self-appraisal (no need to prove one’s own worth).	Inferiority complex (which can manifest as a feeling of superiority) “pushes” one toward success achievement to prove superiority above others.
Interpersonal attitude	Kind inner attitude to others (even in the case of conflicting interests).	Rivalry attitude – the urge to overcome others is a dominant tendency. Treating others as rivals.
People perception	Perceiving individuals based on their values as people. Achievements and successes are not a basis to evaluate a person.	People perception dominated by “better-worse” categories (envy toward “better,” disregard for “worse”).
Emotionality in interpersonal relations	Low hostility level.	Tendency to feel hostility and ascribe hostile tendencies to others.
	Feeling of gratefulness.	Feeling of harm.
	Low anxiety level.	High anxiety level.
	High level of basic trust in people and life.	Low-level of basic trust.
Experiencing self in relations to other people	Feelings of community, unity, harmony with others. Strong sense of bonds with others.	Feelings of isolation, separation from others, alienation. Weak sense of bonds with others.

Adler (1938/2011) believes that the successful completion of life tasks and ability to solve life problems depends on community feeling. A person high in community feeling makes friends easily, is interested in matters that are important for humanity, and is also involved in work for others and being useful for others. High community feeling is also a source of a sense of meaning and of feeling valuable. Community feeling is then beneficial both for the individual and for society. A lack of community feeling, on the other hand, is the basis for many human problems and psychological disorders. It may result in social anxiety, difficulties in cooperation, excessive shyness, distrust, pessimism, feelings of guilt, lust for power, the tendency to enjoy others’ failures, hate, vanity, and demanding attitude toward others (Adler, 1938/2011). These descriptions of high and low community feeling suggest that the latter may be manifested in two main forms: domination/power and fear/isolation. Adler himself did not make an explicit distinction between the two different subtypes of people low in community feeling, but, as prepared by us and shown in Table 1, his descriptions of the characteristics of people low in community feeling provide the basis for two different kinds of anti-community tendencies. First, a more expansive tendency that is manifested by overcoming and dominating others (e.g., in dominant life motivation aspect) and second, a more anxious tendency that is manifested more defensively and by isolating the self from others (e.g., in the aspect of self-experiencing in relation to others). Similar conclusions stem from recent research by Kałużna-Wielobób (2017), who operationalized Adler’s community feeling for empirical research and can be considered a post-Adler approach.

On the basis of Adler’s concept of community feeling and incorporating observations from her psychotherapeutic practice, Kałużna-Wielobób (2017) created an experimental version of the Community Feeling Questionnaire (CFQ), made up of 65 items, which was used to examine 585 adults aged 20–65. A pool of items included in the CFQ was formulated on the basis of Adler’s community feeling characteristics. Then, Item Cluster Analyses (iclust) were performed as an alternative to factor analyses in order to reduce data complexity and to attempt to identify homogeneous subgroupings (Revelle, 2016). This method led to the identification of three clusters. Items indicative of high community feeling created a single cluster, but items indicative of low community feeling created two separate clusters. Therefore, there is initial empirical evidence supporting a differentiation between two types of low community feeling: anti-community domination and anti-community isolation, which differ qualitatively both from high community feeling and from each other.

These three clusters point to three community orientations and can be characterized in the following way that combines Adler’s (1938/2011, 2005) theoretical description and Kałużna-Wielobób (2017) empirical results. First, a pro-community orientation is characterized by the motivation for a common good, a sense of meaning resulting from participating in actions for the common good, a feeling of unity with others, a harmonious group cooperation capacity, kindness toward other people, a focus on working on the quality of relationships with people (perfecting relations) and a tendency to experience gratitude. Second, anti-community domination is characterized by a tendency to dominate and overcome others, a tendency to show one is better than others, a rivalry attitude, a lack in feeling harmony with others, perceiving people via categories “better-worse,” a focus on own benefits without taking the common good into account, hostility and the tendency to ascribe hostility to others. Third, anti-community isolation is characterized by a lack of community feeling, a feeling of isolation and separation from others, the tendency to experience anxiety and tension in groups of people, withdrawal and low self-esteem (inferiority complex).

### Differences Between Constructs: Community Feeling and Communion – Agency

Community feeling in the post-Adler approach (Kałużna-Wielobób, 2017) is not identical to the dimension of communion from the concept of agency-communion popular in social psychology (e.g., Helgeson, 1994; Wojciszke and Abele, 2008; Wojciszke et al., 2009; Abele et al., 2016). Although they are related, the distinctiveness of community feeling and communion can be seen in the theoretical context of both concepts. Below we present a comparison between community feeling and the concept of communion-agency in Wojciszke and Abele’s approach (Wojciszke and Abele, 2008; Wojciszke et al., 2009; Abele et al., 2016), which took into account earlier agency – communion concepts (e.g., Bakan, 1966; Wiggins, 1991; Helgeson, 1994). The main differences can be found in the following areas:

#### What Aspect or Level of Human Functioning a Given Construct Refers to

In the light of the dominant interpretation of communion and agency, they refer to modes of social cognition or behavioral characteristics of interpersonal functioning (Abele and Wojciszke, 2014). A community feeling is a personality disposition related to the dominant motivation of the individual and a sense of bond with a broadly understood community of people.

#### Whether Having a Given Feature Is Beneficial for the Individual or for Others

Communion is characterized as beneficial mainly for others, and agency as favorable mainly for the individual (Abele and Wojciszke, 2014). In a post-Adlerian approach, high community feeling is beneficial for both the others/group and the individual, while low community feeling is unfavorable both for the others/group, and the individual. Acting for the common good implies action that is beneficial to others as well as oneself. Empirical results show that high pro-community orientation (as opposed to anti-community orientations) is associated with high well-being, basic hope and positive affect, as well as lower neuroticism and anxiety (Kałużna-Wielobób, 2017).

#### Structure of the Construct: Single or Bipolar

A low community feeling is not only associated with lack of motivation for the common good, but is also associated with another dynamic motivational force: the desire to show one’s superiority over others. Thus, the negative pole of community feeling is not only passive (lack of pro-community motivation), but it is also dynamic and active (anti-community motivation: striving to defeat others). In contrast, there is no negative pole in the concept of communion – only a higher or lower level of communion.

#### Relationship With Self-Esteem

Studies show that while a high level of agency is associated with high self-esteem, a high level of communion is not. In a series of 12 studies, on different groups, different nationalities, and using different self-esteem measures, agency proved to be an important predictor of self-esteem in all studies, and communion in none of the studies (Wojciszke et al., 2011). According to Adler, low community feeling (or lack thereof) has its origin in the inferiority complex. The desire to beat others, to gain a dominant position or to obtain individual benefits is an attempt to compensate for low self-esteem. Research has shown that community feeling is associated with self-esteem: pro-community orientation is associated positively, and anti-community orientations are associated negatively [with this relationship being stronger in the case of anti-community isolation, and weaker in the case of anti-community domination (Kałużna-Wielobób, 2017)].

Preliminary studies of the correlation between community feeling (measured by the first version of the CFQ) and agency – communion (measured by Agency and Communion Scales – Wojciszke and Szlendak, 2010) have shown that these constructs are related, but not identical. A pro-community orientation proved to be positively correlated with communion (*r* = 0.62), unmitigated communion (*r* = 0.36) and agency (*r* = 0.30) and negatively with unmitigated agency (*r* = −0.36). Anti-community domination is positively related to unmitigated agency (*r* = 0.50) and negatively to communion (*r* = −0.45) and unmitigated communion (*r* = −0.28). Anti-community isolation is negatively correlated with agency (*r* = −0.53) and communion (*r* = −0.36) (Kałużna-Wielobób, 2017).

To sum up, Adler’s (1938/2011, 2005) considerations and some preliminary research (Kałużna-Wielobób, 2017) have shown that two anti-community orientations (anti-community domination and anti-community isolation) can be distinguished at the negative pole of community feeling. Interestingly, these two anti-community orientations seem to correspond closely with the two forms of narcissism: grandiose narcissism and vulnerable narcissism. Hence the question arises as to how well grandiose narcissism and vulnerable narcissism can be mapped onto the two anti-community orientations.

### Narcissism

In various contexts, the term narcissism refers to the developmental phase, personality traits, and the personality disorder. In the DSM-5 (American Psychiatric Association, 2013), a narcissistic personality is characterized by: a fixed pattern of own grandiosity (visible in fantasies and behavior) manifested throughout life, the need for admiration from others, and a lack of empathy. NPD syndrome (*Narcissistic Personality Disorder*) is diagnosed by the presence of at least five of the following criteria: (1) exaggerated self-esteem; (2) fantasizing about unlimited successes; (3) the conviction of own uncommonness and uniqueness, the possibility of being fully understood only by exceptional people and the conviction that it is worth associating only with people or institutions of a special status; (4) requiring excessive admiration; (5) the expectation of being treated in a special way and submitting others to those expectations; (6) exploitation of others; (7) lack of empathy; (8) jealousy toward others or the belief that others envy them; (9) arrogance and haughty behaviors or attitudes.

Since the concept of narcissism derives from psychoanalytic theories, it is worth looking at how narcissism is formulated using this approach. In addition to primary narcissism, which is a normal phase of child development, Freud discussed pathological secondary narcissism, in which libido due to injury is focused mainly on oneself instead of referring to external objects. Secondary narcissism is the nucleus of the psychotic structure and it can also lead to depression. According to Kernberg (2010), the key to narcissism is regulation of self-worth.

Traditionally, the concept of narcissism referred to the psychopathology of personality. In newer psychological literature, however, the more popular approach is to treat narcissism as a subclinical phenomenon that can appear in two forms: grandiose and vulnerable (Wink, 1991; Pincus et al., 2009; Miller et al., 2012; Krizan and Herlache, 2017). Vulnerable narcissism has usually been interpreted as dysfunctional narcissism because of the hostility (malevolence) directed toward the self and others and because of its positive correlations with maladaptive factors such as neuroticism, anxiety, passive aggression, distrust, hostility, avoidance, shyness, and maladaptive time perspectives (negative past and fatalistic present). Furthermore, vulnerable narcissism has negative correlations with self-esteem and well-being. In contrast, although the current formulation in DSM-5 rather refers to grandiose narcissism, it is sometimes interpreted as normal or subclinical, because it correlates with both negative traits, such as aggressiveness or domination, and positive ones such as assertiveness, self-confidence, high self-esteem and well-being (Wink, 1991; Hendin and Cheek, 1997; Miller et al., 2011, 2017; Thomas et al., 2012; Back et al., 2013; Brown et al., 2016; Zajenkowski et al., 2016; Krizan and Herlache, 2017; Rogoza et al., 2018).

In initial studies, the two forms of narcissism were found to be unrelated (Wink, 1991; Hendin and Cheek, 1997). This has been confirmed by subsequent research in the field of personality psychology (Wright and Edershile, 2017). At the same time, clinical psychology research (Pincus and Lukowitsky, 2010) has indicated that people with narcissistic personality disorder have co-occurring grandiose and vulnerable narcissism states. Further studies (Jauk and Kaufman, 2018) have confirmed their independence for the general population, whereas people high on grandiose narcissism might display both (grandiose and vulnerable) aspects. Common to high-intensity grandiose narcissism and vulnerable narcissism is antagonism (Jauk and Kaufman, 2018; Weiss et al., 2019).

Back et al. (2013) specified two strategies of grandiose narcissism: admiration and rivalry. Both strategies serve to maintain the grandiose self-image, but the admiration strategy can be adaptive because, in order to achieve this goal, the individual takes actions to gain the admiration and acceptance of others. These activities are usually socially positive, accompanied by high self-esteem, and the positive response of other people to this strategy. The rivalry strategy, on the other hand, usually turns out to be maladaptive because it involves aggressive behavior, hostility and the devaluation and exploitation of others, which often leads to conflicts (Emmons, 1987; Morf and Rhodewalt, 2001; Rogoza et al., 2016b).

Vulnerable narcissism and the admiration seeking strategy of grandiose narcissism are more removed from each other while the rivalry strategy of grandiose narcissism is located between them and is the closest to self-importance. This was confirmed by Rogoza et al. (2018), who found that the strategy of admiration seeking was negatively related to vulnerable narcissism, and both positively correlated with the rivalry strategy.

The distinction between grandiose and vulnerable narcissism is also considered by the recently developed Narcissism Spectrum Model (Krizan and Herlache, 2017). In this model, narcissism as a personality trait (of varying intensity) and narcissistic personality disorder are found at various points on a narcissism continuum in which narcissistic disorder is an extreme form of narcissistic personality (see also Krueger et al., 2005). The core of narcissism is a sense of self-importance, that is, an excessive focus on the self, high self-esteem and the belief that one’s own needs and goals are more important than the needs and goals of others (Krizan and Herlache, 2017). Depending on other characteristics (for example, approach – avoidance orientation) narcissism takes the grandiose or vulnerable form (grandiose narcissism is approach-oriented whereas vulnerable narcissism is avoidance-oriented).

A somewhat similar distinction is found in the trifurcated model (Weiss et al., 2019). In this model, antagonism connects grandiose and vulnerable narcissism. However, grandiose narcissism also takes the form of agentic extraversion and vulnerable narcissism is close to neuroticism. In this model, agentic aspects of grandiose narcissism coincide with narcissistic admiration – both are adaptive forms of narcissism. Antagonistic aspects include narcissistic rivalry and some aspects of vulnerable narcissism. But vulnerable narcissism also includes neurotic aspects (Back, 2018).

The integration of various approaches to narcissism was made using the Circumplex of Personality Metatraits (Strus and Cieciuch, 2017) – a model used to integrate various personality concepts. The obtained results confirmed assumptions of the Narcissism Spectrum Model (Krizan and Herlache, 2017) that entitlement and self-importance is the core of both narcissism types. In the CPM model, self-importance coincides with the Alpha-Minus/Disinhibition, which represents antagonism toward people, norms, and obligations (Rogoza et al., 2019).

### Research Aims

The theoretical considerations and preliminary research on community feeling led to a distinction between two forms of anti-community orientation: anti-community domination and anti-community isolation. As we presented above, analysis of their theoretical content suggests that they correspond to two facets of narcissism: grandiose and vulnerable. It is worth noting that both community feeling and narcissism are constructs of psychoanalytical origin, proposed by the founder of the psychoanalytical paradigm (Freud and Adler). On the other hand, both community feeling and narcissism are conceptualized and operationalized in current psychology, which enables the relations between these constructs to be tested in the individual differences framework. The aim of our study was to use this opportunity and empirically verify the theoretically predicted relations between two psychoanalytical classic concepts: community feeling and narcissism.

Specifically, based on the considerations presented above we hypothesized that:

(1) Both types of anti-community orientation are negatively related to the pro-community orientation.
(2) Narcissistic rivalry as a facet of grandiose narcissism is positively related to narcissistic admiration (another facet of grandiose narcissism) and vulnerable narcissism.
(3) All aspects of narcissism are negatively related to community feeling (pro-community orientation).
(4) Grandiose narcissism is positively related to anti-community domination (and the relationships will be stronger for narcissistic rivalry than narcissistic admiration) while vulnerable narcissism is positively related to anti-community isolation.

**FIGURE 1:**
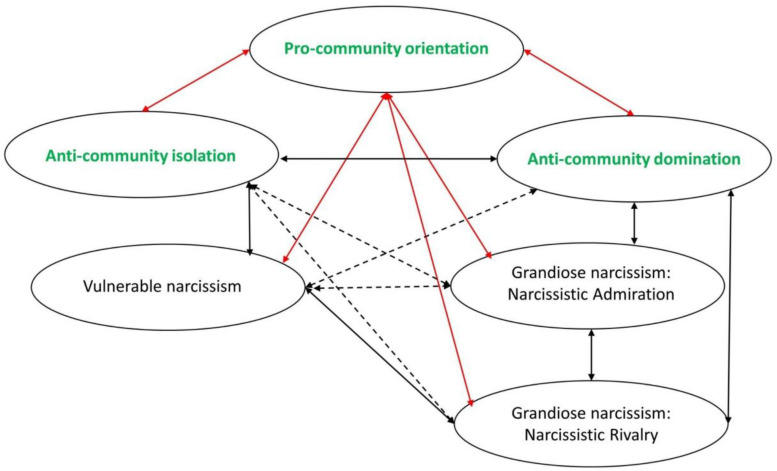
Model of relations between community feeling and narcissism. Red line, expected negative relations; black, expected positive relations; dotted lines, no specific expectations.

All hypotheses will be tested together in a model presented in Figure 1.

## Materials and Methods

### Participants

The sample consisted of 520 participants, including 386 women and 134 men with ages ranging from 18 to 62 (M = 21.37; SD = 4.31). The questionnaires were distributed to 562 people, but 42 people did not fill in the questionnaires completely and therefore were dropped from the analyses. Participants were Polish university students of the following disciplines: special education, social work, English, German, and French philology, psychology, history, speech therapy, cultural management, national security, management, administration, IT, mechanics and machine construction, and power engineering.

### Study Procedure

The study was conducted in 2018 in Polish universities. After consultation with the lecturers, at the beginning of the classes, students were given questionnaires (in paper form) with a request to complete them. Participation in the study was voluntary and involved no incentives for the participants. The questionnaires were filled in anonymously. Students filled them out immediately, during the classes, and after filling them in, the sheets were collected.

### Measures

#### Community Feeling Questionnaire – Revised

We used a revised version of the CFQ (Kałużna-Wielobób, 2017). The CFQ-Revised used in the present study has 46 items: pro-community orientation scale has 18 items, anti-community domination has 14 items and, anti-community isolation also has 14 items. Principal axis factoring (PAF) followed by varimax rotation was conducted on the final 46 items which led to the extraction of a three-factor solution accounting for 44.0% of the variance. These three factors had eigenvalues of 10.11, 6.23, and 3.88 and the following three factors had much lower eigenvalues (i.e., 1.88, 1.35, and 1.18, respectively), which supported the three-factor structure of the measure. After varimax rotation, the first factor (anti-community isolation) explained 15.2% of the variance, while the second factor (community feeling – pro-community orientation) explained 12.8%, and the third factor (anti-community domination) explained 12.3% of the variance. The Cronbach’s alpha reliability coefficients were: pro-community orientation: α = 0.89, anti-community domination: α = 0.90, anti-community isolation: α = 0.92. Content descriptions of the CFQ scales are provided in more detail above and the final set of the CFQ items can be found in the Appendix Table A1 together with a full factor matrix of the items’ PAF analysis. The questionnaire is available at the link https://osf.io/6euyq/.

#### Narcissistic Admiration and Rivalry Questionnaire (NARQ) (Back et al., 2013; Polish adaptation: Rogoza et al., 2016a)

The NARQ measures grandiose narcissism, understood as a personality trait, and consists of 18 items measured on a 6-point Likert scale (1 = *completely disagree* to 6 = *completely agree*). These items make up two subscales representing the two strategies of maintaining a grandiose self-image: admiration and rivalry. The Cronbach’s alphas of the NARQ subscales were: admiration: α = 0.85; rivalry: α = 0.86.

#### Hypersensitive Narcissism Scale (HSNS; Hendin and Cheek, 1997)

The HSNS is a brief, unidimensional measure of vulnerable narcissism (Hendin and Cheek, 1997; Krizan and Herlache, 2017), which covers hypersensitivity, anxiety, withdrawal, and feelings of being neglected. The HSNS items were selected on the basis of the correlation with the MMPI’s narcissistic personality disorder scale. The questionnaire includes 10 items (α = 0.77.), measured on a 6-point Likert scale (1 = *completely disagree* to 6 = *completely agree*).

## Results

### Descriptive Statistics and Correlations Between Variables

Table 2 presents the basic descriptive statistics of the scales used in the study.

**TABLE 2:** Descriptive statistics and results of r-Pearson correlation analyses between community feeling and narcissism.

	**α**	**M**	**SD**	**1**	**2**	**3**	**4**	**5**	**6**
Pro-community orientation (1)	0.89	4.01	0.70	1					
Anti-community domination (2)	0.90	2.49	0.82	−0.41**	1				
Anti-community isolation (3)	0.92	3.19	0.99	−0.17**	0.22**	1			
Grandiose narcissism									
Narcissistic admiration (4)	0.85	3.24	0.91	−0.03	0.47**	−0.32**	1		
Narcissistic rivalry (5)	0.86	2.41	0.93	−0.44**	0.68**	0.24**	0.38**	1	
Vulnerable narcissism (6)	0.77	3.27	0.77	−0.36**	0.47**	0.55**	0.09*	0.44**	1

**p < 0.01; *p < 0.05.

The positive pole of community feeling (pro-community orientation) was negatively correlated both with grandiose narcissism – rivalry, and with vulnerable narcissism. We did not find a significant relationship between community feeling and the admiration strategy of grandiose narcissism. Anti-community domination correlated positively with all forms of narcissism, but, consistent with our expectations, it correlated most strongly with the rivalry strategy of grandiose narcissism. Anti-community isolation, as predicted, correlated most strongly and positively with vulnerable narcissism, as well as negatively with grandiose admiration.

Regarding gender differences: men have a higher level of anti-community domination and of grandiose narcissism (both: rivalry and admiration) than women. There are no other significant gender differences. The results are presented in Table 3.

**TABLE 3:** Differences between men and women – results of Student *t*-test.

	**Men**		**Women**		
	**M**	**SD**	**M**	**SD**	***t***
Pro-community orientation	3.94	0.69	4.03	0.71	–1.31
Anti-community domination	2.87	0.82	2.38	0.79	6.07**
Anti-community isolation	3.24	0.96	3.18	1.01	0.59
Grandiose narcissism					
Narcissistic admiration	3.38	1.04	3.14	0.92	2.45*
Narcissistic rivalry	2.83	1.02	2.29	0.88	5.88**
Vulnerable narcissism	3.29	0.80	3.26	0.76	0.38

**p < 0.01; *p < 0.05.

### The Model of Relations Between Community Feeling and the Two Forms of Narcissism

The theoretical model presented in Figure 1 was tested using a confirmatory factor analysis (CFA) in Mplus 8 (Muthén and Muthén, 2012). While evaluating the model fit, we followed the cutoff criteria proposed in the literature (Hu and Bentler, 1999; Marsh et al., 2004). In the measurement part of the SEM, three parcels were introduced constructing each latent variable and items were designated to the parcels randomly (Little et al., 2002; Bandalos, 2008). In the first step, the CFA model with the obtained parcels for each questionnaire was tested. The results were as follows: (1) The model of CFQ: df = 24, χ^2^ = 75.67, RMSEA = 0.064 (0.048–0.081); CFI = 0.985, SRMR = 0.036; (2) The model for NARQ: df = 8, χ^2^ = 11.85, RMSEA = 0.030 (0.000–0.064); CFI = 0.998, SRMR = 0.020. In the second step the full SEM model was run.

**FIGURE 2:**
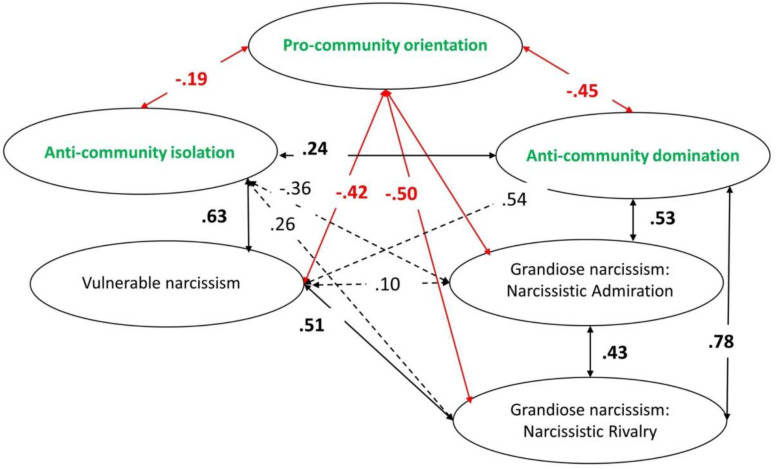
CFA model of relations between community feeling and narcissism. All presented coefficients are significant (*p* < 0.05).

The model presented in Figure 2 obtained a satisfactory fit to the data: χ^2^ = 333.44 (df = 120), CFI = 0.967; RMSEA = 0.058 (0.051–0.066); SRMR = 0.043. As shown, most of the expected relations were confirmed. The only exceptions were the lack of a negative relation between narcissistic admiration and pro-community orientation and (2) a negative relation between narcissistic admiration and anti-community isolation.

## Discussion

In the current article, we argue that community feeling derived from the tradition of Adler is systematically related to narcissism originated in the tradition of Freud. Narcissism has been heavily studied in social, personality, and clinical psychology for decades. Taking into account Adler’s conceptualization of community feeling, one can claim that narcissism can be treated as a deficit in community feeling. Thus, community feeling and narcissism can be treated as two opposing phenomena.

The results of the current study confirmed the hypothesis that anti-community orientations are strongly related to narcissism. Especially anti-community domination is positively related to grandiose narcissism (as measured by NARQ) and anti-community isolation is positively related to vulnerable narcissism (as measured by HSNS).

**FIGURE 3:**
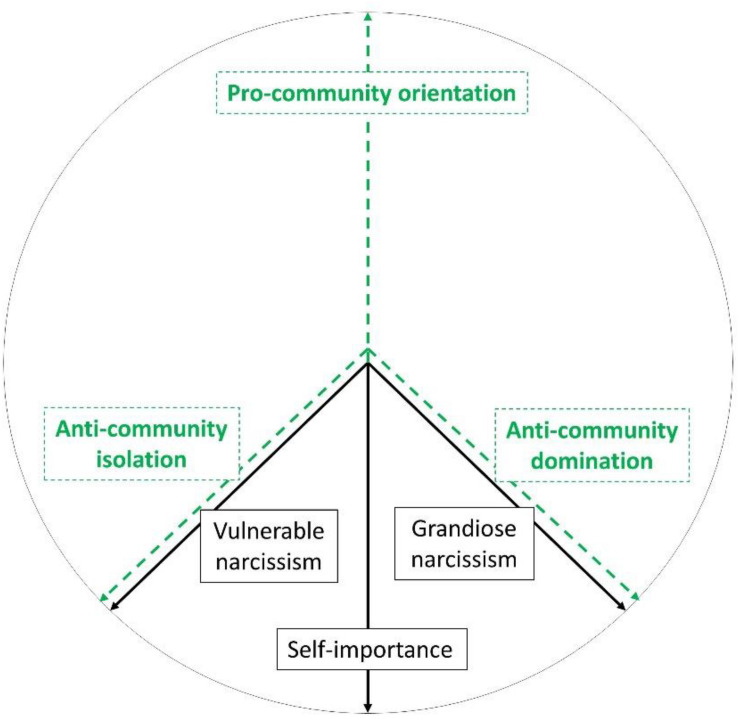
Community feeling as an opposite phenomenon to narcissism.

The relations between community feeling and both aspects of narcissism on the basis of the Spectrum Narcissism Model (Krizan and Herlache, 2017) are presented in Figure 3.

Treating community feeling (pro-community orientation) and narcissism as two opposite psychological phenomena does not mean they are identical or reducible to each other. Describing some constructs as opposing is not rare in psychology. It is especially useful in circular models. An example of that is Schwartz’s (Schwartz et al., 2012) model of values where self-transcendence is treated as opposed (but not reduceable) to self-enhancement and openness to change as opposed (but not reduceable) to conservation. Other examples of circular models with opposing constructs are Wiggins’s Interpersonal Circumplex (Wiggins and Trobst, 1997), and the Circumplex of Personality Metatraits by Strus and Cieciuch (2017). Opposing constructs are possible not only in circular models. A non-circular example is the Light Triad (Kaufman et al., 2019) as opposed to the Dark Triad (Paulhus and Williams, 2002). Such a conceptualization means that, on the one hand, narcissism can be understood in terms of a deficit or an extremely low level of community feeling, and on the other hand, narcissism can contribute to understanding the meaning of anti-community orientations.

The above conclusions, however, mainly concern maladaptive forms of narcissism. Our research has not confirmed the hypothesis of a negative relationship between pro-community orientation and one form of grandiose narcissism: narcissistic admiration. It can be explained by the fact that a narcissistic admiration strategy can have an adaptive function (Back et al., 2013). Research (Rogoza et al., 2019) has shown that an admiration strategy is more remote from the narcissism main axis than other forms of narcissism. Perhaps in the case of people with moderately high self-importance, high community feeling protects against undertaking a rivalry (antagonistic) strategy. Hence, they choose the admiration strategy instead of the rivalry strategy. However, a greater intensity of self-importance already combined with antagonism (or neurotic narcissistic tendency) are related to low community feeling. To better understand the relationship between narcissistic admiration and community feeling, future research should also take into account communion narcissism (Gebauer et al., 2012).

The strength of the article lies in it taking a step toward the integration of two psychological concepts originally derived from two different classical psychoanalytical traditions. At the same time, this has been done by conducting research with tools enabling their integration into contemporary personality concepts. It is worth noting that the concept of narcissism is derived from the works of Freud (1914/1955) and his continuators (in particular Kernberg and Kohut). Those concepts mainly focused on maladaptive narcissism forms (dark sides of narcissism). The concept of community feeling proposed by Adler comes from a positive, adaptive quality of personality, however, it also describes the negative consequences of lacking community feeling. Therefore, the current study contributes to the integration of psychological constructs distinguished by two great psychoanalytic traditions.

The results after some replications can also have a practical application. In particular, showing the connection between maladaptive forms of narcissism and community feeling can be used in the psychotherapy of people with narcissistic personality disorder. According to Adler’s assumption, community feeling, although it is a relatively stable disposition, is formed in the first years of life and can also be potentially developed in later life periods. Therefore, one can expect that supporting the development of community feeling will help in narcissistic personality disorder psychotherapy.

### Limitations and Further Directions

Our study is not free of limitations. First, there are some measurement limitations. The validity of the HSNS (as a measurement of vulnerable narcissism) has been discussed and is considered controversial (Cheek et al., 2013). Additionally, this tool does not allow a distinction to be made between antagonistic narcissism and neurotic narcissism. Further studies are necessary to check the validity of CFQ-revised also in terms of relations with other external variables. Moreover, it would also be worthwhile to examine community feeling with other methods than just the self-description method. Especially other-report would be valuable.

Second, there are some construct limitations that need to be taken into account in future research. Community feeling measured with the CFQ has to be distinguished in empirical results from related constructs like communion (from the communion-agency concept; Wojciszke and Abele, 2008; Wojciszke et al., 2009), agreeableness from the Five Factor Model of Personality (McCrae and Costa, 2003) and stability from the Two-Factor Model of Personality (Cieciuch and Strus, 2017) or the Circumplex of Personality Metatraits (Strus and Cieciuch, 2017). It would also be advisable to conduct research on the relationship between community feeling and narcissistic personality disorder. Future research has to solve the issue of the relationship between community feeling and adaptive narcissism forms like agentic narcissism, including testing the hypothesis that in the case of people with moderately high self-importance, a pro-community orientation will protect against choosing the rivalry (antagonistic) strategy.

## Data Availability Statement

The data are available at the link https://osf.io/6euyq/.

## Ethics Statement

Ethical review and approval was not required for the study on human participants in accordance with the local legislation and institutional requirements. Written informed consent for participation was not required for this study in accordance with the national legislation and the institutional requirements.

## Author Contributions

AK-W was the author of the first version of the operationalization of the Adlerian community feeling (developed the Community Feeling Questionnaire) and collected the data. AK-W and WS developed the revised version of the Community Feeling Questionnaire. AK-W, WS, and JC designed the study. WS and JC ran the statistical analyses. AK-W in collaboration with WS wrote the manuscript. JC commented on the draft of the manuscript and helped to improve it to the published version. All authors read and approved the submitted version.

## Conflict of Interest

The authors declare that the research was conducted in the absence of any commercial or financial relationships that could be construed as a potential conflict of interest.

## Figures and Tables

**TABLE A1:** Matrix of rotating factors from exploratory factor analysis (principal axis factoring with varimax rotation) of the Community Feeling Questionnaire – Revised.

**Items**		**Factor**		
		**1**	**2**	**3**
44	*Przebywając w różnych grupach społecznych, często czuję się wyobcowany.* When I am in different social groups, I often feel alienated.	0.796		
18	*W kontaktach społecznych często reaguję wycofaniem.* In social interactions I often react with withdrawal.	0.794		
22	*Mam trudności ze znalezieniem swojego miejsca w grupie ludzi.* I have difficulty finding my place in a group of people.	0.766		
25	*W kontaktach z ludźmi często czuję się niepewny i onieśmielony.* In dealing with people I often feel uncertain and intimidated.	0.756		
2	*Często kiedy przebywam wśród ludzi, czuję się od nich “oddzielony,” samotny, wyizolowany.* Frequently when I am among people, I feel “separated” from them, alone, isolated.	0.732		
36	*W sytuacjach społecznych często czuję się źle.* I often feel bad in social situations.	0.723		
39	*Często czuję się gorszy od innych.* I often feel worse (inferior) than others.	0.718		
12	*Kontaktując się z ludźmi, których nie znam dobrze, czuję się zwykle zdenerwowany i spięty.* When contacting people I do not know well, I usually feel nervous and tense.	0.662		
33	*Często zdarza się, że z czegoś rezygnuję z powodu lęku przed porażką.* It often happens that I give up on something because of the fear of failure.	0.618		
4	*W różnych sytuacjach wymagających współdziałania w grupie trudno mi się odnaleźć i “wpasować.”* In different situations requiring cooperation in a group, I find it difficult blend in and “fit in.”	0.617		
37	*Kiedy poniosę nawet niewielką porażkę, czuję się mało wartościowy i dotkliwie to przeżywam.* When I suffer a small defeat, I feel of little value and I experience it painfully.	0.599		
6	*Często obawiam się, że inni źle mnie ocenią.* I am often afraid that others will judge me badly.	0.588		
40	*Jest grupa ludzi, z którymi mocno się utożsamiam, ale w większości innych grup czuję się wyobcowany*. There is a group of people with whom I strongly identify, but in most other groups I feel alienated.	0.566		
46	*Zdarza się, że rezygnuję z dążenia do tego, na czym mi zależy z obawy, że źle wypadnę w oczach innych ludzi.* Sometimes, I give up on pursuing what I care about because I fear that I will be seen negatively by other people.	0.534		
17	*Chciałbym, żeby to, co robię, służyło kolejnym pokoleniom bez względu na to, czy będą pamiętać moje imię.* I would like what I do to serve future generations, regardless of whether they remember my name.		0.693	
28	*Uważam, że robiąc coś dla innych, działam dla wspólnego dobra.* I believe that by doing something for others, I act for the common good.		0.686	
15	*To, żeby moje działanie przynosiło skutki dobre również dla innych ludzi jest dla mnie ważniejsze niż to, czy zostanę doceniony.* The fact that my action also brings good results for other people is more important to me than whether I will be appreciated or not.		0.630	
8	*Staram się podejmować takie działania, żeby następne pokolenia miały dobre warunki do życia.* I try to undertake such actions so that the next generations have good conditions for living.		0.620	
9	*Ważne jest dla mnie, żeby ludzie, którzy “przyjdą po mnie”, mogli skorzystać z efektów mojej pracy.* It is important for me that people who will be here after me can benefit from the results of my work.		0.620	
30	*Mam poczucie, że działając dla wspólnego dobra, działam również dla siebie.* I feel that acting for the common good, I also act for myself.		0.614	
5	*Uznam moje życie za sensowne i udane, jeśli to, co zrobiłem/robię/zrobię, przyda się innym, niezależnie od tego, czy zostanie to docenione.* I will recognize my life as purposeful and successful if what I did/do will be useful to others, regardless of whether it will be appreciated.		0.587	
31	*Jeśli źle mi się współpracuje z grupą, to koncentruję się na tym, żeby poprawić swoje relacje z ludźmi.* If I do not cooperate well with a group, I focus on improving my relationships with people.		0.564	
3	*Uważam, że kluczem do sukcesu jest współpraca, nawet jeśli wkład pojedynczych jednostek nie jest wówczas tak podkreślany.* I think that cooperation is a key to success, even if the contribution of an individual unit is not very emphasized.		0.510	
1	*Wolę działać dla wspólnego dobra niż dbać o własne korzyści kosztem dobra innych.* I prefer to work for the common good rather than to look to benefit myself at the expense of the good of others.		0.505	
13	*Kiedy mam trudność w kontakcie z kimś z mojej grupy, staram się nad tym pracować.* When I have difficulty in interacting with someone in my group, I try to work on it.		0.489	
11	*Lubię współpracę z innymi ludźmi*. I like cooperating with other people.		0.485	
34	*Reakcje innych osób na to, co robię są dla mnie źródłem informacji o tym, jak polepszyć swoje relacje z ludźmi.* The reactions of others to what I do are a source of information for me on how to improve my relationships with people.		0.483	
42	*Generalnie lubię dzielić się czymś z ludźmi.* Generally, I like to share something with people.		0.478	
26	*Nawet kiedy jestem z jakąś osobę w konflikcie, to mimo tego dobrze jej życzę.* Even when I am in conflict with a person, I still wish them well.		0.457	
16	*Często myślę o tym, że wiele zawdzięczam innym ludziom.* I often think about the fact that I owe a lot to other people.		0.437	
23	*Jestem życzliwie nastawiony do innych ludzi i dobrze życzę nawet tym, których nie lubię.* I am sympathetic to other people and I wish others well even those I do not like.		0.436	
20	*Często odczuwam wdzięczność wobec innych ludzi*. I often feel gratitude toward other people.		0.407	
38	*Marzę o sławie i byciu podziwianym.* I dream about fame and being admired.			0.729
43	*Uznam moje życie za sensowne, jeśli będę pełnić stanowisko związane z władzą nad innymi lub z wysoką pozycją społeczną.* I will consider my life purposeful if I am in a position of power over others or with a high social position.			0.725
27	*Marzę o władzy.* I dream about power.			0.717
24	*Uznam moje życie za udane, jeśli osiągnę sukcesy pokazujące moją wyższość nad innymi.* I will recognize my life as successful if I succeed in showing my superiority over others.			0.697
41	*Często czuję się lepszy od innych.* I often feel better than others (superior over others).			0.688
14	*Sukcesy sprawiają, że czuję się lepszy od innych.* Successes make me feel better than others (superior to others).			0.598
21	*Osiąganie sukcesu czyimś kosztem nie przeszkadza mi w dążeniu do celu.* Achieving success at someone’s expense does not prevent me from pursuing my goal.			0.596
10	*Czasami lekceważę ludzi, którzy niewiele osiągnęli.* Sometimes I disregard people who have not achieved much.			0.586
19	*Nie ma dla mnie znaczenia, czy mój zysk oznacza cudzą stratę.* It does not matter to me whether my gain means someone else’s loss.			0.558
29	*Często widzę w innych ludziach rywali.* I often see rivals in other people.			0.539
45	*Jeżeli nie osiągam swoich celów, to zwykle uważam, że winni są za to inni ludzie.* If I do not achieve my goals, I usually think that other people are to blame.			0.536
7	*Staram się podejmować tylko te działania, w których mogę zatriumfować i okazać się lepszym od innych.* I try to take only those actions in which I can triumph and prove myself better than others.			0.509
32	*W grupie albo jestem “gwiazdą,” albo się od niej dystansuję.* In the group, I am either a “star” or I distance myself from it.			0.440
35	*Osoby, które przekazują mi uwagi krytyczne, działają zwykle na moją szkodę.* People who give me critical remarks usually act to my detriment.			0.354

*Loadings below 0.35 are hidden.*
